# Characterization of Human Bladder Cell Membrane During Cancer Transformation

**DOI:** 10.1007/s00232-015-9770-4

**Published:** 2015-01-09

**Authors:** Izabela Dobrzyńska, Barbara Szachowicz-Petelska, Barbara Darewicz, Zbigniew A. Figaszewski

**Affiliations:** 1 Institute of Chemistry, University in Białystok, Al. Piłsudskiego 11/4, 15-443 Białystok, Poland; 2 Clinical Department of Urology, Medical University of Białystok, Skłodowskiej-Curie 24A, 15-276 Białystok, Poland; 3 Laboratory of Electrochemical Power Sources, Faculty of Chemistry, University of Warsaw, Pasteur St. 1, 02-093 Warsaw, Poland

**Keywords:** Phospholipids, Proteins, Electric charge, Human bladder

## Abstract

Phenomena associated with changes in cell membranes are thought to play an important role in the cancer transformation. We hypothesized that the electrical charge of tumor cells can indirectly represent membrane-based changes that have occurred during cell transformation and may indicate tumor cell status. Here, we describe work showing that phospholipids, proteins content, and electric charge, are all altered in the cell membranes of pT2 stage/grade G3 bladder cancer. Qualitative and quantitative phospholipid composition and the presence of integral membrane proteins were identified using high-performance liquid chromatography. Protein composition was determined using selective hydrolysis of isolated bladder cell membrane proteins and peptide resolution. The surface charge density of human bladder cell membranes was determined using electrophoresis. Our results show that cancer transformation is associated with increased phospholipid levels and a decreased level of integral proteins. Moreover, the process of cancer transformation significantly enhanced changes in the surface charge density of the human bladder cell membrane. In conclusion, this study demonstrates that cell membrane structure and function are modified in bladder cancer cells and that further work in this area is warranted.

## Introduction

In more highly developed countries, bladder cancer is the fourth most common type of cancer in men and the ninth most common cancer in women. More than 50,000 men and 16,000 women are diagnosed with bladder cancer each year. Smoking a risk factor for bladder cancer can only partially explain the higher incidence in men. One other reason is that the androgen receptor, which is much more active in men than it is in women, plays a major part in the development of bladder cancer (Miyamoto et al. 2007).

Tumor cells produce and excrete numerous blood-based factors called tumor biomarkers. Tumor biomarkers exhibit diverse physicochemical properties and can be classed according to several groups: fetal cancer, antigens, carcinogenic antigens, proteins, cell metabolism products, cell growth factors, hormones, enzymes, and isoenzymes (Szachowicz-Petelska et al. 2002). Since biomarkers have to be transported across the cell membrane, their expression is affected by the biophysical properties of membranes, including the membrane, electric charge, and the change in potential between the membrane and its surrounding solution. Electric properties of the membrane are determined by acid–base and complex formation equilibria of membrane and solution components (Szachowicz-Petelska et al. 2012). Most membrane components including proteins and phospholipids are involved in those equilibria.

Phospholipids play two roles in the mammalian cell. They serve as basic structural components of membranes and are also substrates of reactions involved in key regulatory functions. Thus, abnormalities in phospholipid metabolism is one of the major hallmarks of cancer. In fact, the identification of cancer-associated changes in the precursors and catabolites of phospholipids is one way to non-invasively monitor tumor progression and tumor response to conventional and targeted anti-cancer therapies (Podo et al. 2011).

Changes in the structural and electrophysiological characteristics of mammalian cell membranes can affect the activity of membranous proteins that serve as ion channels, transporters, receptors, signal transducers or enzymes (Szachowicz-Petelska et al. 2013).

Therefore, examining the electric charge of a tumor cell membrane may reveal a lot of information about the relative state of that cell’s phospholipids and protein content. This work tests the hypothesis that the electrical charge of tumor cells can indirectly represent membrane-based changes that have occurred during cell transformation, and may indicate tumor cell status. We explored this hypothesis by documenting the changes in phospholipid content (by high-performance liquid chromatography, HPLC), protein content (by selective hydrolysis and peptide resolution), and surface charge density (by electrophoresis) of human bladder cell membranes isolated from tumor- and non-tumor-containing tissue samples.

## Materials and Methods

Tissue samples were obtained from eight patients (seven men and one woman) who underwent radical cystectomy for the treatment of muscle-invasive, high-grade urothelial carcinoma. All patients had lymph node involvement at the time of surgery but no distant metastases. The age of patients ranged from 58 to 78 years old. Tumor samples and control bladder tissue (macroscopically unchanged mucosa) were collected from each patient after bladder removal with line incision. All control, non-neoplastic tissues were pathologically examined to exclude for the presence of cell dysplasia.

### Electrochemical Methods

In order to determine surface charge density of the cell membrane, bladder tissue samples were digested with trypsin and separated cells were collected. The electrophoretic mobility of these cells, at a given pH was measured using the Zetasizer Nano ZS apparatus (Malvern Instruments Ltd). The surface charge density was then calculated using the following equation: *σ* = *ηu*/*d*; where *u* is the electrophoretic mobility, *η* in the viscosity of the solution, and *d* is the diffuse layer thickness (Krysiński and Tien 1986). The diffuse layer thickness was calculated using the following equation:

$$ d = \sqrt {\frac{{\varepsilon \cdot \varepsilon_{0} \cdot R \cdot T}}{{2 \cdot F^{2} \cdot I}}} , $$

where *R* is the gas constant, *T* is the temperature, *F* is the Faraday number, *I* is the ionic strength of 0.9 % NaCl, and *ε* and *ε*_o_ are the relative and absolute permittivity constants of the medium (Barrow 1996).

Acidic (*C*_TA_) and basic ($C_{{{\text{TB}}^{ \cdot } }}$) functional group concentrations and their average association constants with hydrogen (*K*_AH_) or hydroxyl ($K_{{{\text{BOH}}^{ \cdot } }}$) ions were determined, as described previously (Dobrzyńska et al. 2006).

### Phospholipid and Protein Isolation and Analysis by High-Performance Liquid Chromatography (HPLC)

Bladder cell membranes were prepared using the differential centrifugation method, as described by Ipata (1967) and Evansa (1970). Phospholipids were then extracted using chloroform–methanol as described by Folch et al. (1957). Normal phase (NP)-HPLC separations were done using a Merck HPLC system equipped with a pump, an ultraviolet (UV) detector, an analog interface module (D-6000 A) and System Manager software. Phospholipids were separated using a silica gel column, with an acetonitrile/methanol/phosphoric acid (85 %) mixture (130:5:1.5 volume ratio) by isocratic elution at 1 mL/s flow rate and 214 nm wavelength (Dobrzyńska et al. 2005).

**Fig. 1:**
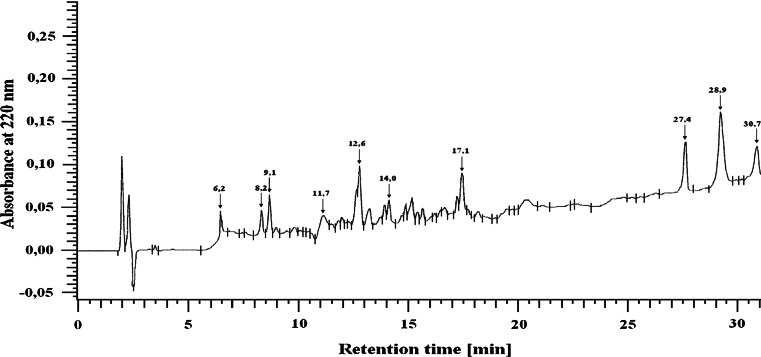
A characteristic example of the separation of peptides from integral membrane proteins from bladder tissue (UV detected at 220 nm)

The membrane protein extracts were hydrolyzed using trypsin, at an enzyme:substrate ratio of 1:25 (Persaud et al. 2000). Following hydrolysis, the peptides were separated by HPLC on a LichroCART RP-18 column 100 A (5 μm, 250 × 4.0 mm) equilibrated with solvent A [0.1 % trifluoroacetic acid (TFA) in H_2_O] and eluted with a linear gradient of 20–100 % solvent B (0.1 % TFA in acetonitrile) using a flow rate of 1 mL/min (Szachowicz-Petelska et al. 2010). A typical separation of the peptide mixture containing integral bladder membrane proteins is provided in Fig. 1.

Isolated peptides were hydrolyzed at various lengths of time (6.2, 8.2, 9.1, 11.7, 12.6, 14, 17.1, 27.4, 28.9, and 30.7 min) using trypsin as described previously (Szachowicz-Petelska et al. 2010). The amino acid separation was performed on a Lichrosorb NH_2_ column 100 A (5 μm, 250 × 4.6 mm). The mobile phase consisted of solvents A (0.01 M KH_2_PO_4_, pH 4.3) and B (a 500:70 mixture of acetonitrile/water). All separations were performed with a 5–50 % gradient of solvent A using a flow rate of 1 mL/min. All of the peptides originated from different groups that consistently contained the following four amino acids: phenylalanine (Phe), tyrosine (Tyr), cysteine (Cys), and lysine (Lys).

### Statistical Methods

The data obtained in this study are expressed as mean ± SD. The data were analyzed using Wilcoxon matched-pairs signed-ranks test from a standard statistical program (SPSS 8.0 PL) for comparisons between control and cancer samples. Values *P* < 0.05 were considered significant.

## Results

### Electric Properties of Non-neoplastic and Neoplastic Bladder Cell Membranes

**Fig. 2:**
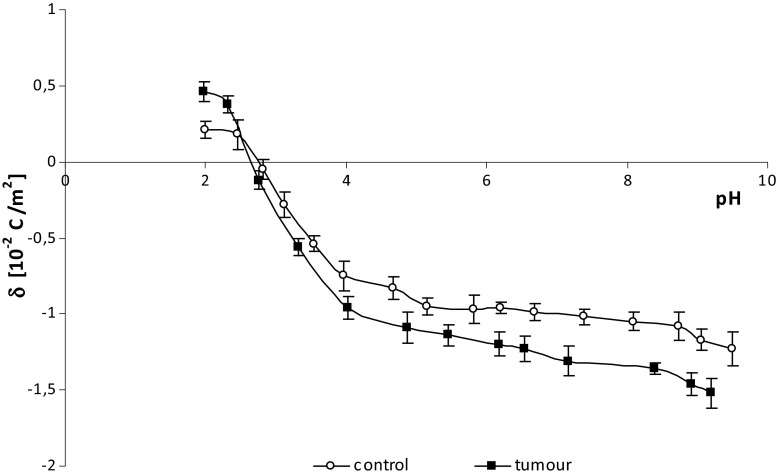
The dependence of surface charge density on pH of non-neoplastic and bladder cancer cells from several patients

The effects of surface charge density on pH are similar between normal and bladder cancer cell membranes (Fig. 2). There is an increase in positive surface charge density at low pH values but only up to a point. Conversely at high pH values, the negative charge of the cells is increased until it too reaches a plateau. Overall, compared to unaffected cells, human bladder cancer cells are more negatively charged at low pH values, and more positively charged at high pH values.

The surface fraction occupied by phospholipids and proteins as well as the surface concentration of acidic groups (*C*_TA_) and the surface concentration of basic groups (*C*_TB_) of non-neoplastic and neoplastic human bladder cell membranes are presented in Table 1. An increase in the surface concentration of both acidic and basic groups was observed in the samples prepared from tissues that contained cancer with respect to non-neoplastic controls. The *C*_TA_, *C*_TB_, and K_BOH_ values of cancer cell membranes were higher than in non-neoplastic cells, while *K*_AH_ decreased in comparison (Table 1).

**Table 1:** *C*_TA_, *C*_TB_, *K*_AH_, and *K*_BOH_ from human bladder membrane containing pT2-staged/G3-graded cancer

Patient no’s	*C*_TA_ (10^−7^ mol/m^2^)	*C*_TB_ (10^−7^ mol/m^2^)	*K*_AH_ (m^3^/mol)	*K*_BOH_ (10^7^ m^3^/mol)
1.				
Control	1.21 ± 0.09	0.21 ± 0.04	42.80 ± 0.93	1.86 ± 0.08
Tumor	1.51 ± 0.10^a^	0.49 ± 0.05^a^	30.16 ± 1.01^a^	2.12 ± 0.10^a^
2.				
Control	1.07 ± 0.08	0.74 ± 0.07	45.18 ± 0.92	2.32 ± 0.10
Tumor	1.51 ± 0.09^a^	1.09 ± 0.08^a^	30.62 ± 1.11^a^	2.68 ± 0.12^a^
3.				
Control	1.41 ± 0.08	0.86 ± 0.05	39.18 ± 1.12	1.86 ± 0.09
Tumor	1.82 ± 0.10^a^	1.03 ± 0.06^a^	28.34 ± 1.09^a^	2.53 ± 0.11^a^
4.				
Control	1.16 ± 0.09	0.93 ± 0.07	38.12 ± 1.10	1.62 ± 0.08
Tumor	1.53 ± 0.11^a^	1.29 ± 0.09^a^	30.22 ± 1.01^a^	2.14 ± 0.10^a^
5.				
Control	1.43 ± 0.12	0.43 ± 0.07	37.48 ± 0.91	2.12 ± 0.09
Tumor	1.79 ± 0.11^a^	0.75 ± 0.08^a^	26.13 ± 1.05^a^	2.36 ± 0.10^a^
6.				
Control	2.12 ± 0.12	0.62 ± 0.07	31.21 ± 0.92	1.95 ± 0.06
Tumor	2.51 ± 0.10^a^	0.91 ± 0.06^a^	27.57 ± 0.84^a^	2.31 ± 0.08^a^
7.				
Control	1.63 ± 0.08	0.42 ± 0.04	45.12 ± 1.11	1.97 ± 0.11
Tumor	2.14 ± 0.09^a^	0.76 ± 0.05^a^	34.43 ± 1.06^a^	2.31 ± 0.10^a^
8.				
Control	1.21 ± 0.07	0.54 ± 0.06	37.42 ± 1.01	1.65 ± 0.06
Tumor	1.72 ± 0.06^a^	0.88 ± 0.07^a^	28.12 ± 0.93^a^	2.14 ± 0.11^a^

Statistically significant differences corresponded to *P* < 0.05

^a^In comparison with control (non-neoplastic tissue)

### The Phospholipid Composition of Non-neoplastic and Neoplastic Bladder Cell Membranes

**Fig. 3:**
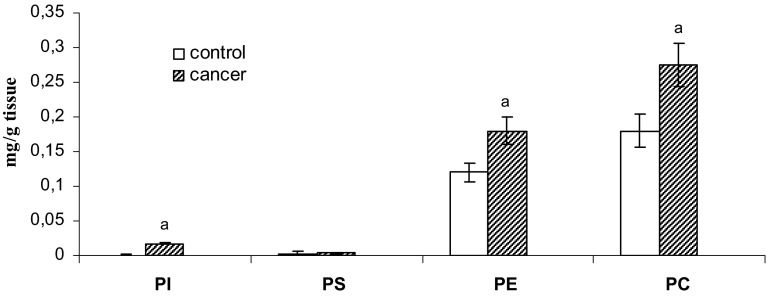
The amounts of cell membrane phospholipids from human bladder tissue containing pT2-staged/G3-graded tissue. Statistically significant differences corresponded to *P* < 0.05. ^a^In comparison with control (non-neoplastic tissue)

Figure 3 shows that the levels of phospholipids in the cell membrane was significantly increased in the cancer-containing samples, as compared to matched non-neoplastic controls, and that the changes observed for samples collected from all eight patients were similar. Specifically, the content of individual phospholipids [phosphatidylinositol (PI), phosphatidylserine (PS), phosphatidylethanoloamine (PE), and phosphatidylcholine (PC)] all increased.

In order to study protein modifications under pathological conditions, both qualitative and quantitative estimations of protein content are necessary. Herein, we used a HPLC-based approach that involved selective hydrolysis of isolated tissue cell membrane proteins to peptides, resolution by chromatography, and determination of the amino acid content [phenylalanine (Phe), tyrosine (Tyr), cysteine (Cys), and lysine (Lys)] in individual peptides.

### Integral Membranous Proteins of Non-neoplastic and Neoplastic Bladder Cells

**Fig. 4:**
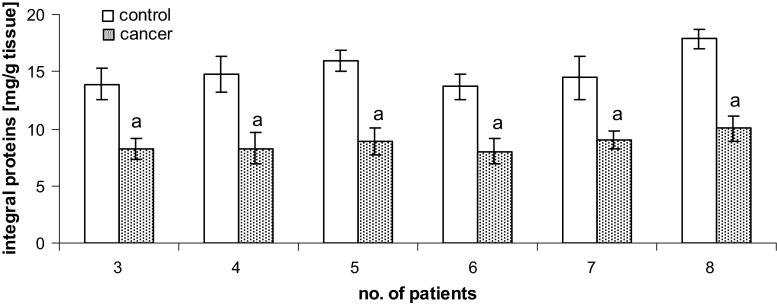
Content of integral membrane proteins isolated from human bladder tissue containing pT2-staged/G3-graded cancer. Statistically significant differences corresponded to *P* < 0.05. ^a^In comparison with control (non-neoplastic tissue)

As seen in Fig. 4, the levels of integral membrane proteins decreased significantly during cancer transformation. We observed a roughly 40 % decrease in integral membrane protein levels for the bladder cells isolated from tissue containing pT2-staged/G3-graded cancer, with respect to matched, non-neoplastic control samples.

**Fig. 5:**
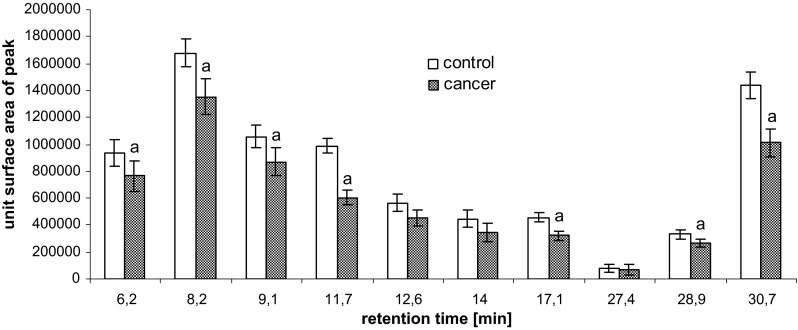
Content of peptides of hydrolyzed cell membranes isolated from human bladder tissue containing pT2-staged/G3-graded cancer. Statistically significant differences corresponded to *P* < 0.05. ^a^In comparison with control (non-neoplastic tissue)

Figure 5 shows average and individual changes in peptide content after the hydrolysis of proteins isolated from cell membranes from the control and tumor samples. Overall, cancer was associated with a decrease in peptide levels, relative to non-neoplastic control samples.

Finally, levels of the Phe, Tyr, Cys, and Lys amino acids were determined for individual peptides following bladder membrane protein hydrolysis. Individually and across an average, the cancer tissues showed a decrease in the amount of individual amino acids, relative to the non-neoplastic control tissues (Table 2).

**Table 2:** Content of the amino acids Phe, Cys, Tyr, and Lys for individual peptides following membrane protein hydrolysis of cell membranes from human bladder tissue containing pT2-staged/G3-graded cancer

Concentration of amino acids (Phe, Tyr, Cys, Lys)							
Amino acid	Peptide fractions	Control	Tumor	Amino acid	Peptide fractions	Control	Tumor
Phe (μg/mg protein)	6.2	2.61 ± 0.53	1.97 ± 0.49	Tyr (ng/mg protein)	6.2	0.26 ± 0.03	0.18 ± 0.02^a^
	8.2	1.34 ± 0.15	1.01 ± 0.15^a^		8.2	0.30 ± 0.04	0.21 ± 0.03^a^
	9.1	1.65 ± 0.22	1.18 ± 0.12^a^		9.1	0.21 ± 0.01	0.13 ± 0.07
	11.7	2.43 ± 0.14	1.58 ± 0.10^a^		11.7	0.24 ± 0.05	0.18 ± 0.04
	12.6	2.73 ± 0.17	2.15 ± 0.10^a^		12.6	0.30 ± 0.02	0.21 ± 0.02^a^
	14	1.69 ± 0.34	1.22 ± 0.14		14	0.18 ± 0.03	0.10 ± 0.02
	17.1	1.71 ± 0.33	1.16 ± 0.22		17.1	0.28 ± 0.05	0.24 ± 0.04
	27.4	1.58 ± 0.14	1.02 ± 0.08^a^		27.4	0.29 ± 0.03	0.19 ± 0.03^a^
	28.9	2.85 ± 0.56	1.83 ± 0.37^a^		28.9	0.19 ± 0.06	0.14 ± 0.05
	30.7	3.04 ± 0.11	2.02 ± 0.13^a^		30.7	0.30 ± 0.02	0.21 ± 0.01^a^
Cys (ng/mg protein)	6.2	40.3 ± 9.38	31.8 ± 7.21	Lys (μg/mg protein)	6.2	9.10 ± 0.52	6.72 ± 0.21^a^
	8.2	60.2 ± 5.12	50.7 ± 3.30^a^		8.2	8.22 ± 1.09	5.92 ± 0.93^a^
	9.1	42.0 ± 2.31	26.2 ± 1.99^a^		9.1	5.88 ± 1.49	3.67 ± 1.26
	11.7	36.2 ± 2.81	22.4 ± 2.50^a^		11.7	3.95 ± 0.44	2.45 ± 0.40^a^
	12.6	33.0 ± 6.07	25.5 ± 5.34		12.6	4.96 ± 0.55	3.24 ± 0.35
	14	27.4 ± 4.96	22.9 ± 4.01		14	9.51 ± 1.05	7.44 ± 1.08
	17.1	37.5 ± 2.68	23.4 ± 4.11^a^		17.1	4.17 ± 0.48	2.70 ± 0.40^a^
	27.4	30.2 ± 2.37	21.6 ± 2.29^a^		27.4	7.12 ± 0.66	5.43 ± 0.55^a^
	28.9	28.4 ± 3.54	20.5 ± 3.09^a^		28.9	4.03 ± 0.61	2.09 ± 0.30^a^
	30.7	36.0 ± 5.11	26.6 ± 4.12^a^		30.7	4.17 ± 0.65	2.61 ± 0.44^a^

Statistically significant differences corresponded to *P* < 0.05

^a^In comparison with control (non-neoplastic tissue)

## Discussion

It is well known that malignant cancer cells differ from their normal counterparts, particularly with respect to their surface membrane properties. One of the most important consequences of these altered surface membrane properties is the manifestation of unusual cell-to-cell interactions, which can affect the prognosis of patients suffering from cancer.

These transformations also result in serious metabolic-track perturbations, which are reflected by changes in the content of phospholipids and proteins in biological membranes. Increased levels of phospholipids (Fig. 3) can increase the concentration of functional acidic (*C*_TA_) and basic (*C*_TB_) groups on the bladder cancer cell membrane (Table 1) and their average association constants with hydrogen (*K*_AH_) and hydroxyl (*K*_BOH_) ions. Indeed, the literature shows that abnormalities in phospholipid metabolism represent major hallmarks of cancer cells ((Podo et al. 2011). Changes in the profiles of aqueous precursors and catabolites of phospholipids in cancer lesions allow for the non-invasive monitoring of tumor progression and tumor response to conventional and targeted anti-cancer therapies (Podo et al. 2011).

An analysis of phospholipid content showed that the levels of phosphatidylcholine and phosphatidylethanolamine are higher than that of other phospholipids in normal human bladder and human bladder cancers (Fig. 3). Physiologically regulated catabolism of phosphatidylcholine generates second messengers and mitogens, such as diacylglycerol, phosphatidic acid, lysophosphatidic acid, lysophosphatidylcholine, and arachidonic acid. Phosphocholine (PC) has also been proposed to be mitogenic, by acting as a mediator of growth factor-induced cell proliferation (Podo et al. 2011). Alterations in the intracellular levels of PC in cancer cells during tumor progression or in response to therapy may reflect a multiplicity of modifications taking place at the genetic, epigenetic, transcriptional, and post-transcriptional levels. An increase in PC, for instance, may derive from enhanced choline kinase expression or activity. Its activation, a critical requirement for induction of DNA synthesis by mitogens and growth factors, is implicated in RAS-dependent and RAS-independent carcinogenesis and tumor progression (Janardhan et al. 2006; Remirez de Molina et al. 2004). Choline kinase has also been proposed to be a prognostic factor in breast, non-small cell lung, and bladder cancers (Hermando et al. 2009). Enhanced levels of phosphatidylethanoloamine derivatives can also be detected in tumors, but their values vary more widely in cells, according to the concentration of ethanolamine in the extracellular medium (Franks et al. 2002).

Increased levels of phospholipids within the bladder cell membrane may in turn lead to a decrease in the levels of other charged molecules on the cell surface, such as integral proteins. The results of our study support this, as they show a decrease in the levels of membranous integral proteins of bladder cancer cells, relative to non-neoplastic cells (Fig. 4). The ultimate consequence of cancer transformation-induced protein modifications can include structural changes, such as aggregation or fragmentation (Gabai et al. 1994; Rantanen et al. 2008), leading to the generation of new functional groups, both acidic and basic. These changes can yield higher negative electric charges at high pH values and lower positive electric charges at low pH values, such as what we observed for the samples collected from cancer-containing bladder tissues.

The oxidative stress induced by cancer transformation has been attributed to changes in the structure and function of bladder cell components, including membrane phospholipids and proteins. All amino acids are susceptible to attack by free radicals, although some of them are more vulnerable than others. Those that are most sensitive to oxidation include aromatic amino acids, such as phenylalanine and tyrosine. As a sulfhydryl amino acid, Cys is also extremely sensitive to free radicals (Kalyanaraman 1995). Reports have shown that the within-protein ratio of cysteine:cystine is altered under oxidizing conditions. The occurrence of these types of reactions would explain the decrease in the amount of amino acids detected in our study (Table 2).

Beside the phospholipids and proteins that have already been discussed in this work, the cell membrane charge is also affected by the fact that sialic acid is a component of glycolipids and glycoproteins. Sialic acid also may influence the surface concentration of *C*_TA_ and *C*_TB_ groups, as well as the association constants with positive and negative groups during cancer transformation. The literature indicates that total sialic acid was significantly higher in samples from bladder cancer patients and that its level correlated with the severity of the cancer (Honglertsakul et al. 2007).

In conclusion, our data suggest that the composition and electrical properties of bladder cancer cell membranes are different from those of non-neoplastic cells. We found that the constants *C*_TA_, *C*_TB_, *K*_AH_, and *K*_BOH_ vary in cancer cells and may be suitable for monitoring the changes caused by cancer transformation. Therefore, an evaluation of the parameters characterizing the membranes and membrane status of cancer cells, and other components of membranes, may be an important consideration in future studies of cancer biology.
